# Supplementary material for: Automatic synchronisation of the cell cycle in budding yeast through closed-loop feedback control
Source: Nat Commun. 2021 Apr 27;12:2452. doi: 10.1038/s41467-021-22689-w (PMC8079375; doi:10.1038/s41467-021-22689-w)
Supplement: Supplementary file 1 — Supplementary Information [file 41467_2021_22689_MOESM1_ESM.pdf]

Supplementary Information

Automatic synchronisation of the cell cycle in budding yeast through closed-loop feedback control

Perrino et al.

SUPPLEMENTARY FIGURES

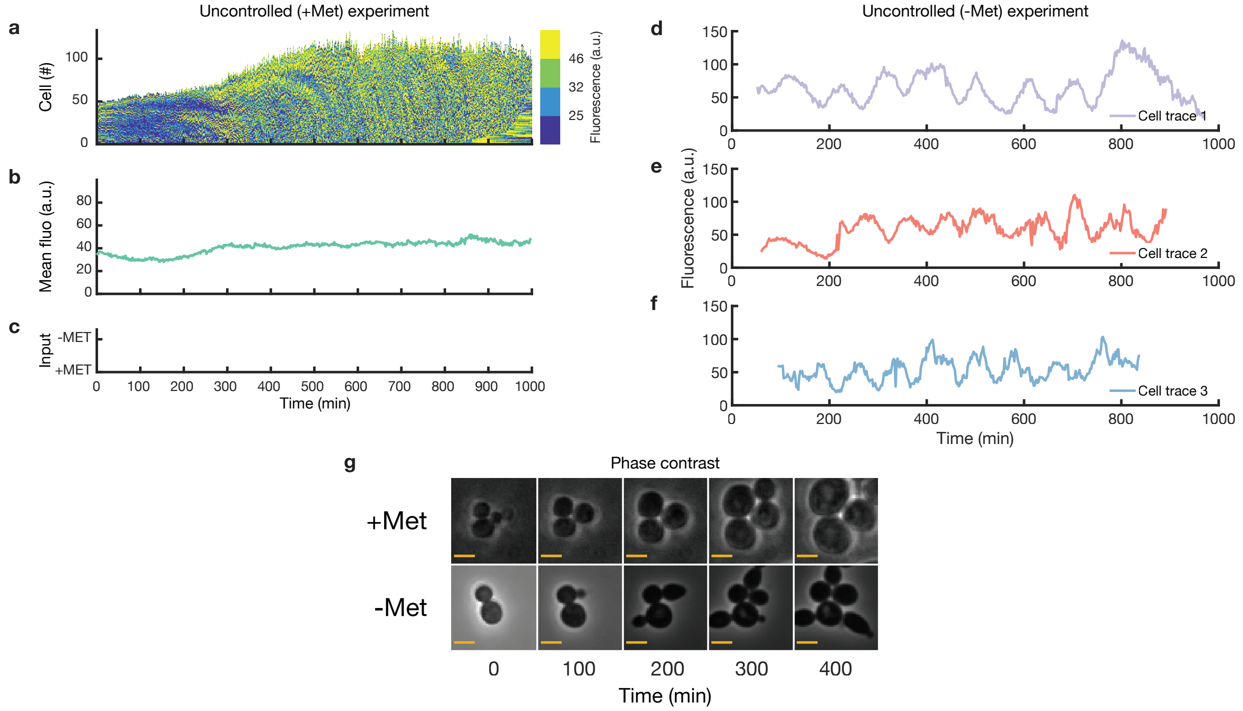

Supplementary Figure 1. Experimental characterisation of the non-cycling strain. a-c, Experimental characterisation of the non-cycling yeast strain grown in the automated microfluidics platform in methionine-rich medium (+MET). **a**, The number of cells and the distribution of YFP fluorescence intensity in the population over time. Fluorescence values are binned into 4 colours, corresponding to the quartiles, for clarity of visualisation. **b**, Average YFP fluorescence intensity in the cell population. **c**, Growth medium delivered to the cells as a function of time: +MET methionine-rich medium, -MET: methionine-depleted medium. **d-f**, YFP fluorescence intensity measured in three representative cells grown in methionine-depleted medium. **g**, Representative phase contrast images from two independent time-lapse experiments of cells grown in methionine-rich medium (top) and methionine-depleted medium (bottom) at the indicated time points. Scale bar, 5 μm.

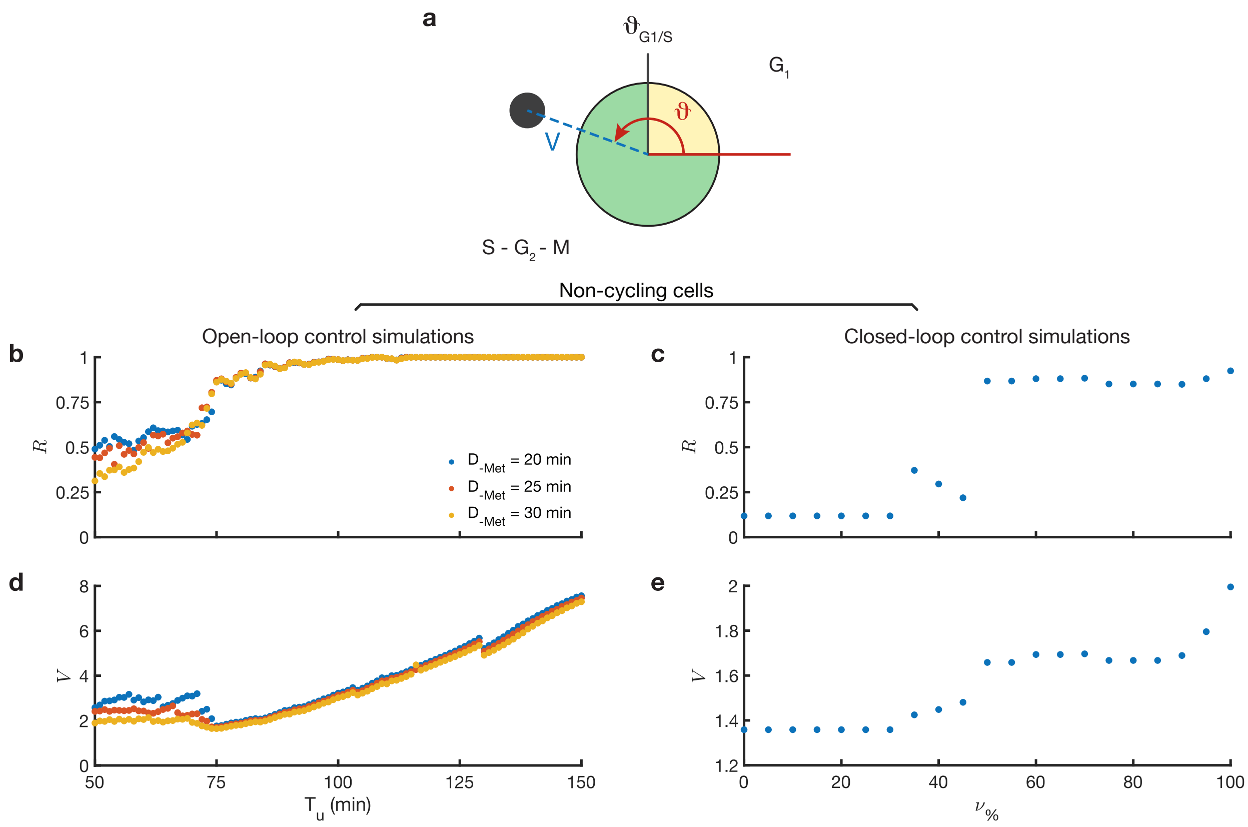

Supplementary Figure 2. Mathematical modelling and simulation of the non-cycling strain. a, Graphical representation of the mathematical model of the cell cycle. The black circle represents a cell evolving in $\mathbb{R}^2$. The cell cycle phase ϑ corresponds to the angle between the red line and the dashed blue line. The length of the dashed blue line corresponds to the cell volume. The yellow shaded sector corresponds to the G_1 phase, while the green shaded sector corresponds to the S–G_2–M phases. The cell cycle phase evolves counter clockwise. **b**, **d**, Time-average of the mean phase coherence R and time-average of the volume growth V for increasing values of the period T_u and for three different pulse duration D_{-Met} computed from numerical simulations of a population of cells in response to periodic stimulation with -MET pulses. **c**, **e**, Time-average of the mean phase coherence R and time-average of the volume growth V for increasing threshold values ($\nu_{\%}$) of the stop&go controller computed from numerical simulations of a population of non-cycling cells.

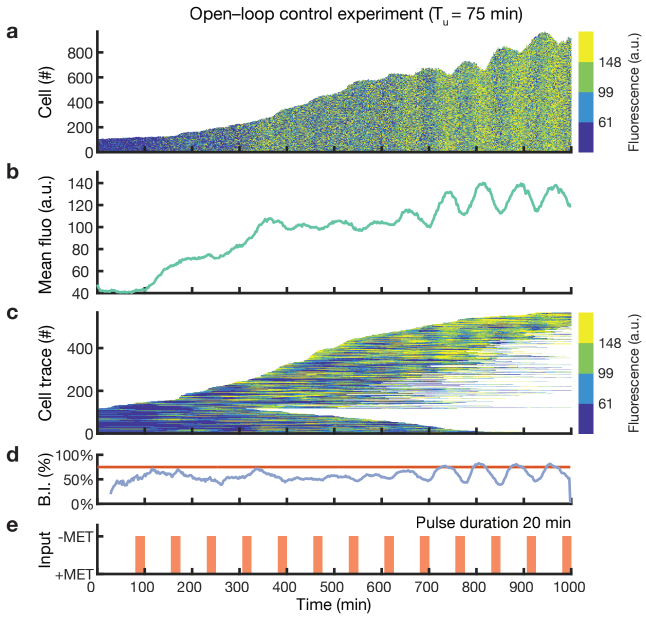

Supplementary Figure 3. Additional *in vivo* open-loop control experiment in the non-cycling yeast strain with a shorter -MET pulse duration. Non-cycling yeast strain cells grown in the automated microfluidics platform in response to alternating pulses of methionine-rich (+MET) and methionine-depleted (-MET) medium with period T_u = 75 min and pulse duration D_{-Met} = 20 min. **a**, The number of cells and the distribution of YFP fluorescence intensity in the population over time. Fluorescence values are binned into 4 colours, corresponding to the quartiles, for clarity of visualisation. **b**, Average YFP fluorescence intensity in the cell population. **c**, Single-cell fluorescence traces over time. Each horizontal line corresponds to one cell. Each line starts when the cell is first detected and ends when the cell exits the field of view. The number of tracked cells does not correspond to the total number of cells as only cells tracked for longer than 300 min are shown. **d**, Budding index (blue) reporting the percentage of cells in the budding phase (S-G_2-M) computed from the estimated cell cycle phases. The red line denotes the expected value of the budding index in the case of a totally desynchronised cell population. **e**, Growth medium delivered to the cells as a function of time: +MET methionine-rich medium, -MET: methionine-depleted medium.

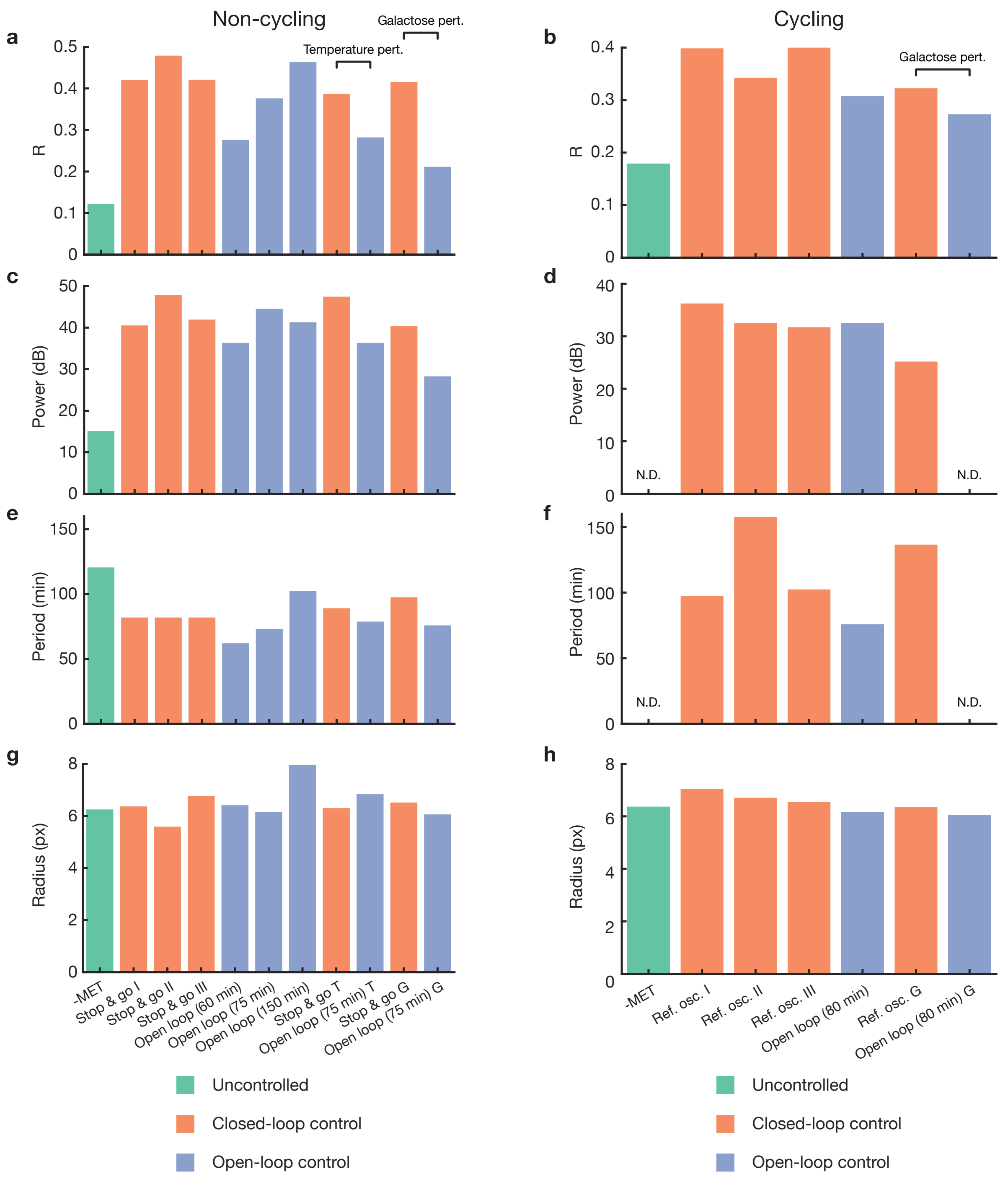

Supplementary Figure 4. Quantification of cell cycle synchronisation and cell size. a, **b**, Time-average of the mean phase coherence R for all the experiments performed in the manuscript. The time-average was computed between 330 and 530 min for the non-cycling strain, and between 100 and 600 min for the cycling strain. **c**, **d**, Amplitude of the leading peak of the power spectrum of the population averaged YFP fluorescence intensity for the non-cycling (**c**) and the cycling (**d**) strains. *N.D.*: not determined – when the average YFP fluorescence intensity is not oscillatory and thus no leading peak can be found (Methods). **e**, **f**, Period of the power spectrum corresponding to the leading peak for the non-cycling (**c**) and the cycling (**d**) strains. **g**, **h**, Time-average of the mean cell radius for the non-cycling (**g**) and the cycling (**h**) strain. T: temperature shifted to 27°C. G: cells grown in Galactose.

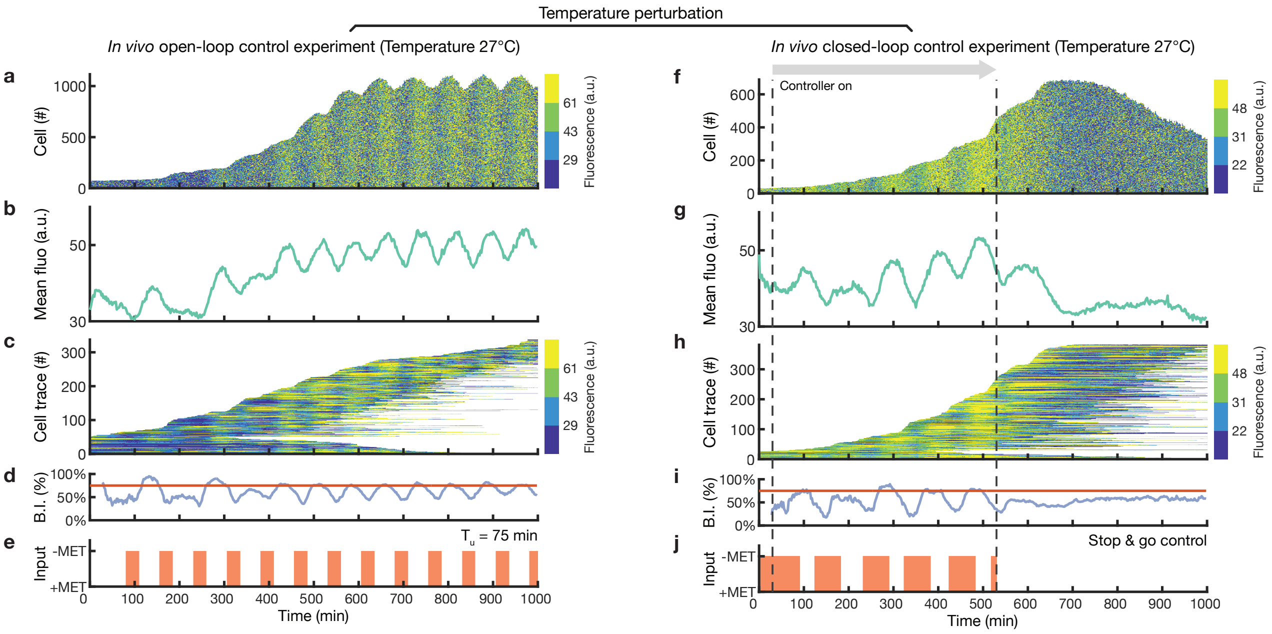

Supplementary Figure 5. Closed-loop stop&go control of the non-cycling strain is robust to temperature perturbation. Temperature was set to 27°C, rather than the nominal 30°C, to assess the robustness of the open-loop and closed-loop control strategies in perturbed environmental conditions. **a-e**, Open-loop control experiment: cells were stimulated with periodic -MET pulses of period T_u = 75 min and pulse duration D_{-Met} = 30 min. **f-j**, Closed-loop control experiment: an initial calibration phase of 30 min was required to set up the phase estimation algorithm. Dashed lines indicate the start and the end of the control experiment, after which cells are grown in methionine-rich medium. **a**, **f**, The number of cells and the distribution of YFP fluorescence intensity in the population over time. Fluorescence values are binned into 4 colours, corresponding to the quartiles, for clarity of visualisation. **b**, **g**, Average YFP fluorescence intensity in the cell population. **c**, **h**, Single-cell fluorescence traces over time. Each horizontal line corresponds to one cell. Each line starts when the cell is first detected and ends when the cell exits the field of view. The number of tracked cells does not correspond to the total number of cells as only cells tracked for longer than 300 min are shown. **d**, **i**, Budding index (blue) reporting the percentage of cells in the budding phase (S-G_2-M) computed from the estimated cell cycle phases. The red line denotes the expected value of the budding index in the case of a totally desynchronised cell population. **e**, **j**, Growth medium delivered to the cells as a function of time: +MET methionine-rich medium, -MET: methionine-depleted medium.

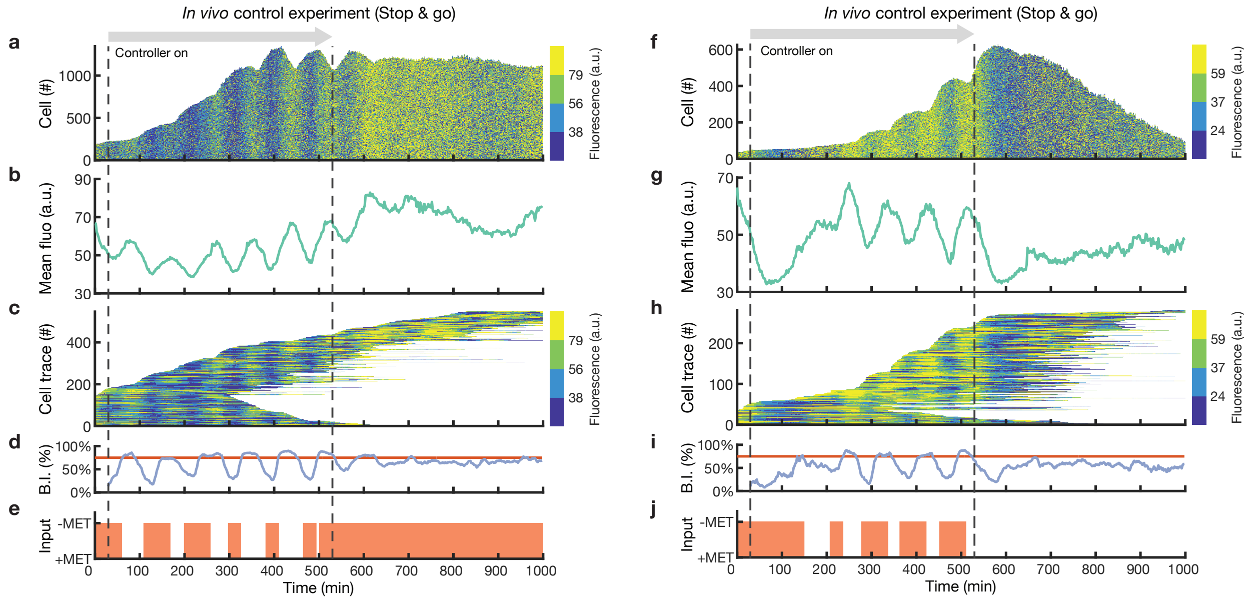

Supplementary Figure 6. Closed-loop stop&go control experiments in the non-cycling yeast strain. An initial calibration phase of 30 min was required to set up the phase estimation algorithm. Dashed lines indicate the start and the end of the control experiment, after which cells are grown either in methionine-depleted medium (**a**-**e**) or methionine-rich medium (**f**-**j**). **a**, **f**, The number of cells and the distribution of YFP fluorescence intensity in the population over time. Fluorescence values are binned into 4 colours, corresponding to the quartiles, for clarity of visualisation. **b**, **g**, Average YFP fluorescence intensity in the cell population. **c**, **h**, Single-cell fluorescence traces over time. Each horizontal line corresponds to one cell. Each line starts when the cell is first detected and ends when the cell exits the field of view. The number of tracked cells does not correspond to the total number of cells as only cells tracked for longer than 300 min are shown. **d**, **i**, Budding index (blue) reporting the percentage of cells in the budding phase (S-G_2-M) computed from the estimated cell cycle phases. The red line denotes the expected value of the budding index in the case of a totally desynchronised cell population. **e**, **j**, Growth medium delivered to the cells as a function of time: +MET methionine-rich medium, -MET: methionine-depleted medium.

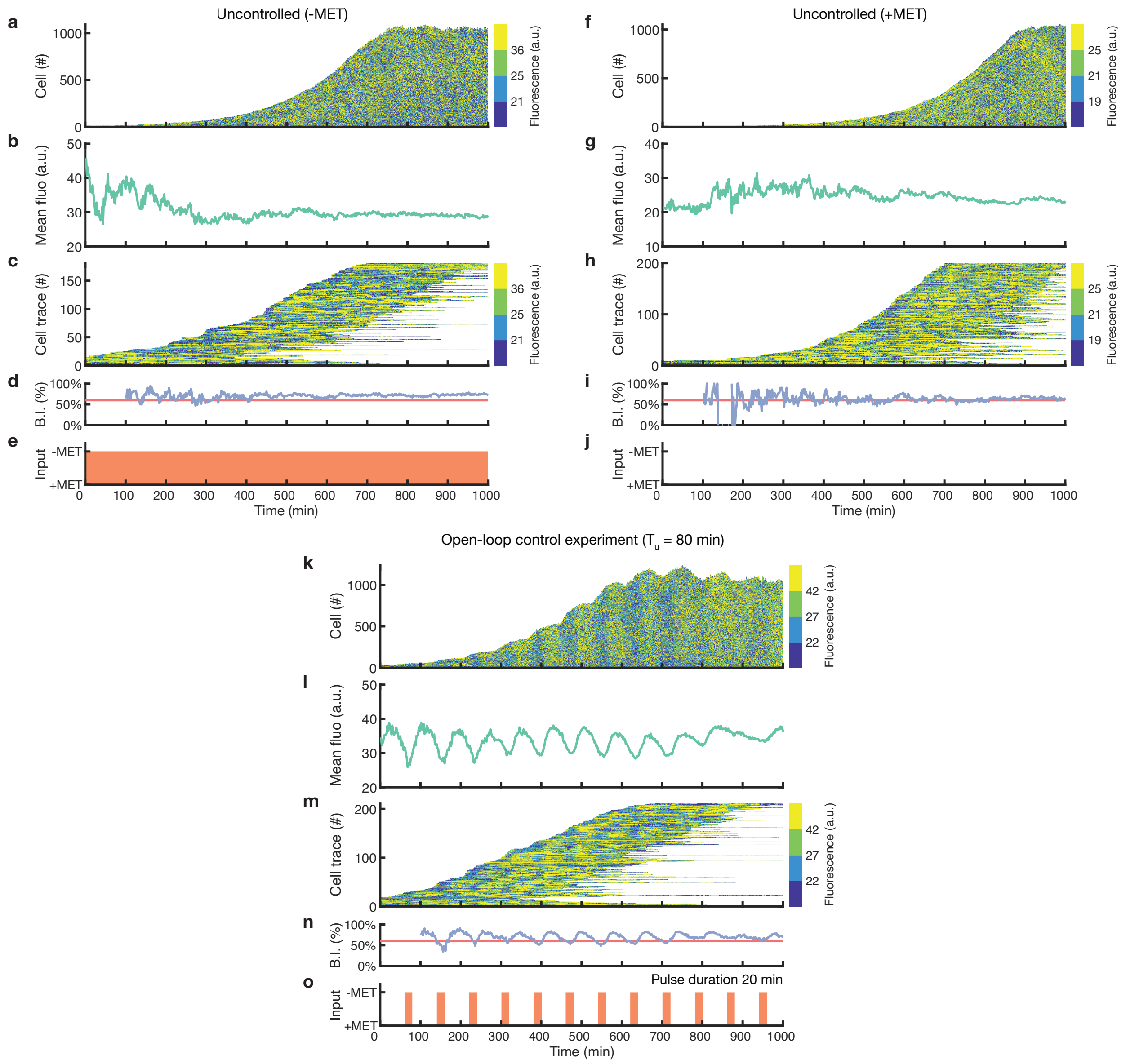

Supplementary Figure 7. Characterisation and open-loop control of the cycling yeast strain. Experiments with cycling yeast strain cells grown in the automated microfluidics platform in three different conditions: (**a-e**) methionine-depleted medium (-MET); (**f-j**) methionine-rich medium (+MET); (**k-o**) alternating pulses of methionine-rich (+MET) and methionine-depleted (-MET) medium with a period T_u = 80 min. Duration of -MET pulse was set to 20 min. **a**, **f**, **k**, The number of cells and the distribution of YFP fluorescence intensity in the population over time. Fluorescence values are binned into 4 colours, corresponding to the quartiles, for clarity of visualisation. **b**, **g**, **l**, Average YFP fluorescence intensity in the cell population. **c**, **h**, **m**, Single-cell fluorescence traces over time. Each horizontal line corresponds to one cell. Each line starts when the cell is first detected and ends when the cell exits the field of view. The number of tracked cells does not correspond to the total number of cells as only cells tracked for longer than 300 min are shown. **d**, **i**, **n**, Budding index (blue) reporting the percentage of cells in the budding phase (S-G_2-M) computed from the estimated cell cycle phases. The red line denotes the expected value of the budding index in the case of a totally desynchronised cell population. **e**, **j**, **o**, Growth medium delivered to the cells as a function of time: +MET methionine-rich medium, -MET: methionine-depleted medium.

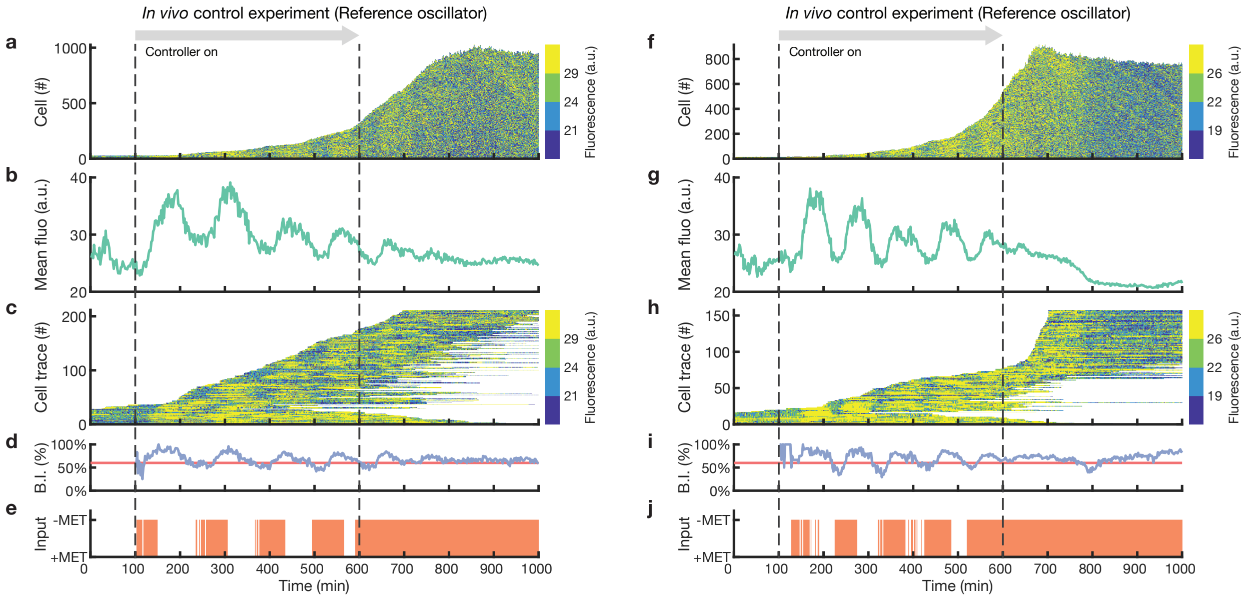

Supplementary Figure 8. Closed-loop reference oscillator control experiments in the cycling yeast strain. Experimental implementation of the reference oscillator control strategy. An initial calibration phase of 100 min is required to set up the phase estimation algorithm. Dashed lines indicate the start and the end of the control experiment, after which cells are grown in methionine-depleted medium. **a**, **f**, The number of cells and the distribution of YFP fluorescence intensity in the population over time. Fluorescence values are binned into 4 colours, corresponding to the quartiles, for clarity of visualisation. **b**, **g**, Average YFP fluorescence intensity in the cell population. **c**, **h**, Single-cell fluorescence traces over time. Each horizontal line corresponds to one cell. Each line starts when the cell is first detected and ends when the cell exits the field of view. The number of tracked cells does not correspond to the total number of cells as only cells tracked for longer than 300 min are shown. **d**, **i**, Budding index (blue) reporting the percentage of cells in the budding phase (S-G_2-M) computed from estimated cell cycle phases. Red line denotes the expected value of the budding index in the case of a totally desynchronised cell population. **e**, **j**, Growth medium delivered to the cells as a function of time: +MET methionine-rich medium, -MET: methionine-depleted medium.

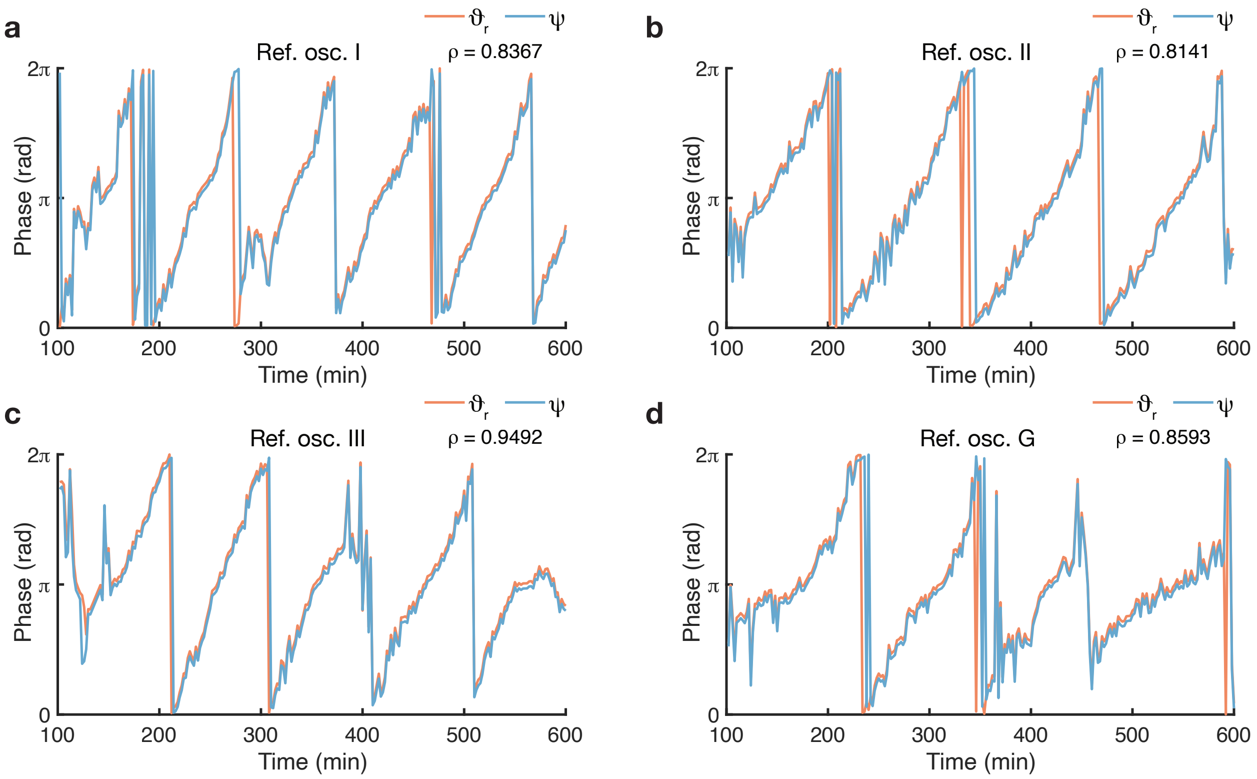

Supplementary Figure 9. Correlation between the phase ϑ_r of the reference oscillator and mean phase ψ of the Kuramoto order parameter in the reference oscillator control experiments. a-**d**, Time-series of the reference oscillator phase ϑ_r (red) and the mean phase ψ of the cell population (blue) for the four experiments performed in the manuscript using the reference oscillator control strategy. The mean phase ψ was computed as the argument of the Kuramoto order parameter (Methods). ρ is the Spearman's rank correlation coefficient. **a**-**c**, Experiments performed in nominal conditions. **d**, Experiment performed in perturbated conditions (galactose used as carbon source).

SUPPLEMENTARY TABLES

Supplementary Table 1. Yeast strains used in this study. All strains are W303 congenic.

Strain	Description	Genotype	Source
SJR14a4d	Non-cycling strain	cln1Δ, cln2Δ::CLN2p-Venus::TRP1, cln3Δ::LEU2, trp1Δ::TRP1::MET3p-CLN2, HTB2-mCherry::HIS5	Ref.[1]
GC84-35B	Background of cycling strain	cln3Δ::LEU2, CDC10-YFP::LEU2, WHI5-GFP::KanMx, TRP1::MET3p-CLN2	Ref.[2]
yDdB028	Cycling strain	cln3Δ::LEU2, CDC10-YFP::LEU2, WHI5-GFP::KanMx, TRP1::MET3p-CLN2, HTB2-mCherry::natNT2	This study

Supplementary Table 2. Plasmids used in this study.

Plasmid	Type	Source
pRS41N	Replicative	Euroscarf; ref.[3]
pRS41N-GAP-CYC	Replicative	C. Wilson
pRS41N-GAP-HTB2-mCherry-CYC	Replicative	This study

Supplementary Table 3. Primers used in this study.

Primer	Description	Sequence
FW HTB2-mCherry	HTB2-mCherry amplification (XBaI tail)	gctctagaATGTCCTCTGCCGCCGA
REV HTB2-mCHerry	HTB2-mCherry amplification (EcoRI tail)	ggaattcctacttgtacagctcgtccatgcc
FW HR	Homologous recombination (cycling strain)	acaacaacttactctacaactaATGTCCTCTGC CGCCGAAAAGAAACCAG
RV HR	Homologous recombination (cycling strain)	gtcaaggagggtattctgggcctccatgtcgccact aataaaaagaaaacatGACTAAATCACAATA CCTAGTGAGTGAC

SUPPLEMENTARY NOTES

Supplementary Note 1. Derivation of the reference oscillator strategy. The reference oscillator strategy was derived from the one proposed by Bai and Wen to asymptotically synchronise a homogeneous population of phase oscillators using a single (i.e., common) control input[4]. To derive the reference oscillator strategy, we made a series of assumptions that allowed us to adapt the state feedback control law proposed by Bai and Wen[4] to solve the cell cycle synchronisation problem in a population of cycling yeast cells using an asymmetrically bounded input. Firstly, we neglected the dynamics associated to cell growth and cell division. Such an assumption implies a constant number of cycling cells in the population. Secondly, we assumed that the cell cycle dynamics in each cell is well approximated by a first-order reduced phase dynamics determined by a phase response curve (PRC). Such an assumption is well in line with our mathematical framework (see Modelling section in Methods). Let N denote the number of cycling cells in the cell population. Then, the cell cycle dynamics of each cell is represented by an ODE model that describes the cell cycle phase evolution in time:

$$\frac{d}{dt}\vartheta = \omega + z(\vartheta)\, u\,, \tag{1}$$

where $\omega \in \mathbb{R}_+$ is the natural frequency of the cell cycle oscillator, $\vartheta \in \mathbb{S}^1$ is the 2π-periodic cell cycle phase on the unit circle, $u \in \{u_{OFF}, u_{ON}\}$ is the external trigger input, and $z : \mathbb{S}^1 \to \mathbb{R}_+$ is the 2π-periodic PRC function that models the linear response of the cell cycle phase ϑ to the input u. Note that the proposed control input u is asymmetrically bounded *a priori*. Indeed, in our modelling framework $u_{OFF} = 0$ (i.e., when the cell is fed with methionine-rich medium), while $u_{ON} = 1$ (i.e., when the cell is fed with methionine-depleted medium). Also, in our modelling framework, we mathematically described the PRC function as:

$$z(\vartheta) = \begin{cases} \omega_z & \text{if } 0 \le \vartheta < \vartheta_{G_1/S} \\ 0 & \text{if } \vartheta_{G_1/S} \le \vartheta < 2\pi \end{cases}, \tag{2}$$

where $\omega_z \in \mathbb{R}_+$ is the angular velocity added to the cell cycle phase dynamics when $u = 1$ (i.e., $u = u_{ON}$), and $\vartheta_{G_1/S}$ is the cell cycle phase value at the G_1 to S transition. We then derived a state feedback control law for an asymmetrically bounded input as presented in the Section III of Bai and Wen[4]. We introduced a reference oscillator modelled by the first-order reduced phase dynamics:

$$\frac{d}{dt}\vartheta_r = \omega_r + \gamma \sum_{m=1}^{N} \sin(\vartheta_m - \vartheta_r)\ , \tag{3}$$

where $\omega_r \in \mathbb{R}$ is the natural frequency of the reference oscillator, $\vartheta_r \in \mathbb{S}^1$ is the 2π-periodic cell cycle phase of the reference oscillator, $\gamma \in \mathbb{R}_+$ is the coupling strength, and ϑ_m is the cell cycle phase of the *m*-th cell in the population. Let $\boldsymbol{\vartheta} = [\vartheta_1, \ldots, \vartheta_N]^T$ and $h(\boldsymbol{\vartheta}) = [z(\vartheta_1), \ldots, z(\vartheta_N)]^T$. Let $\delta_m = \vartheta_m - \vartheta_r$ define the phase error associated to the *m*-th cell. Let $\boldsymbol{\delta} = [\delta_1, \ldots, \delta_N]^T$ and $\sin(\boldsymbol{\delta}) = [\sin(\delta_1), \ldots, \sin(\delta_n)]^T$. We thus considered the state feedback control law [refer to Section III in Bai and Wen[4]]:

$$u = -u_{ON}\,\rho_u\left(\sin(\boldsymbol{\delta})^T\, h(\boldsymbol{\vartheta})\right), \tag{4}$$

where the continuous function $\rho_u(\cdot): \mathbb{R} \rightarrow [-1,0]$ satisfies $\rho_u(s) = 0$, $\forall s \geq 0$ and $-s \geq \rho_u(s)\, s > 0$, $\forall s < 0$. Being that $\|\sin(\boldsymbol{\delta})^T\, h(\boldsymbol{\vartheta})\| \leq N\,\omega_z$, then we could choose:

$$\rho_u(s) = \begin{cases} 0 & \text{if } s \geq 0 \\ -\dfrac{s}{N\,\omega_Z} & \text{if } s < 0 \end{cases}, \tag{5}$$

where $s = \sin(\boldsymbol{\delta})^T\, h(\boldsymbol{\vartheta})$. However, $u \in \{0,1\}$ is a bang-bang control input, thus we devised a robust realisation of the continuous function $\rho_u(\cdot)$, that is:

$$\rho_u(s) = \begin{cases} 0 & \text{if } s \geq 0 \\ -1 & \text{if } s < 0 \end{cases}, \tag{6}$$

where $s = \sin(\boldsymbol{\delta})^T\, h(\boldsymbol{\vartheta})$. The previous function leads to the bang-bang state feedback control:

$$u = \begin{cases} 0 & \text{if } s \geq 0 \\ 1 & \text{if } s < 0 \end{cases}, \tag{7}$$

where $s = \sin(\boldsymbol{\delta})^T\, h(\boldsymbol{\vartheta})$. However,

$$\sin(\boldsymbol{\delta})^T\, h(\boldsymbol{\vartheta}) = \sum_{m=1}^{N} \sin(\vartheta_m - \vartheta_r)\; z(\vartheta_m) = \sum_{m=1}^{N} \mathrm{a_m} \sin(\vartheta_m - \vartheta_r)\; \omega_z = \omega_z \sum_{m=1}^{N} \mathrm{a_m} \sin(\vartheta_m - \vartheta_r)\;, \tag{8}$$

where $\mathrm{a_m} \in \{0,1\}$ is a coefficient denoting if the *m*-th cell is in the G_1 phase, that is:

$$a_m = \begin{cases} 1 & \text{if } 0 \leq \vartheta_m < \vartheta_{G_1/S} \\ 0 & \text{if } \vartheta_{G_1/S} \leq \vartheta_m < 2\pi \end{cases}. \tag{9}$$

Thus, we obtained that

$$s = \sin(\boldsymbol{\delta})^T\, h(\boldsymbol{\vartheta}) \geq 0 \Leftrightarrow \omega_z \sum_{m=1}^{N} \mathrm{a_m} \sin(\vartheta_m - \vartheta_r) \geq 0\,. \tag{10}$$

Being $\omega_z > 0$, then the previous inequality holds also when

$$\sum_{m=1}^{N} \mathrm{a_m} \sin(\vartheta_m - \vartheta_r) \geq 0\,. \tag{11}$$

Finally, we obtained the bang-bang state feedback control law as reported in the Methods:

$$u = \begin{cases} u_{OFF} = 0, & \text{if } \sum_{m=1}^{N} \mathrm{a_m}\ \sin(\vartheta_m - \vartheta_r) \geq 0 \\ u_{ON} = 1, & \text{if } \sum_{m=1}^{N} \mathrm{a_m}\ \sin(\vartheta_m - \vartheta_r) < 0 \end{cases}\,. \tag{12}$$

Such a control law, under the previous assumptions, guarantees that the cell cycle phases converge asymptotically towards the reference phase according the Theorem 2 stated in Bai and Wen[4].

SUPPLEMENTARY REFERENCES

1. Rahi, S. J., Pecani, K., Ondracka, A., Oikonomou, C. & Cross, F. R. The CDK-APC/C Oscillator Predominantly Entrains Periodic Cell-Cycle Transcription. *Cell* **165**, 475–487 (2016).
2. Charvin, G., Cross, F. R. & Siggia, E. D. Forced periodic expression of G1 cyclins phase-locks the budding yeast cell cycle. *Proc. Natl. Acad. Sci. U. S. A.* **106**, 6632–6637 (2009).
3. Taxis, C. & Knop, M. System of centromeric, episomal, and integrative vectors based on drug resistance markers for Saccharomyces cerevisiae. *Biotechniques* **40**, 73–78 (2006).
4. Bai, H. & Wen, J. T. Asymptotic Synchronization of Phase Oscillators With a Single Input. *IEEE Trans. Automat. Contr.* **64**, 1611–1618 (2019).
