# Supplementary material for: Automatic synchronisation of the cell cycle in budding yeast through closed-loop feedback control
Source: Nat Commun. 2021 Apr 27;12:2452. doi: 10.1038/s41467-021-22689-w (PMC8079375; doi:10.1038/s41467-021-22689-w)
Supplement: Supplementary file 3 — Description of Additional Supplementary Files [file 41467_2021_22689_MOESM3_ESM.pdf]

Title: Supplementary Movie 1.
Description: Experimental characterisation of the non-cycling strain in methionine-depleted medium. Related to Fig. 2a-e.

Title: Supplementary Movie 2.
Description: Open-loop control of non-cycling cells with forcing period T_u = 75 min and pulse duration D_{-Met} = 30 min. Related to Fig. 2k-o.

Title: Supplementary Movie 3.
Description: Open-loop control of non-cycling cells with forcing period T_u = 150 min and pulse duration D_{-Met} = 30 min. Related to Fig. 2p-t.

Title: Supplementary Movie 4.
Description: Closed-loop control of non-cycling cells. Related to Fig. 3f-j.

Title: Supplementary Movie 5.
Description: Experimental characterisation of the cycling strain in methionine-supplemented medium. Related to Supplementary Fig. 7f-j.

Title: Supplementary Movie 6.
Description: Closed-loop control of cycling cells using the refence oscillator strategy. Related to Fig. 4f-j.

Title: Supplementary Data 1.
Description: Data used to estimate the nominal cell cycle period of the cycling strain.
