# Supplementary figures and images for: Automatic synchronisation of the cell cycle in budding yeast through closed-loop feedback control
Source: Nat Commun. 2021 Apr 27;12:2452. doi: 10.1038/s41467-021-22689-w (PMC8079375; doi:10.1038/s41467-021-22689-w)

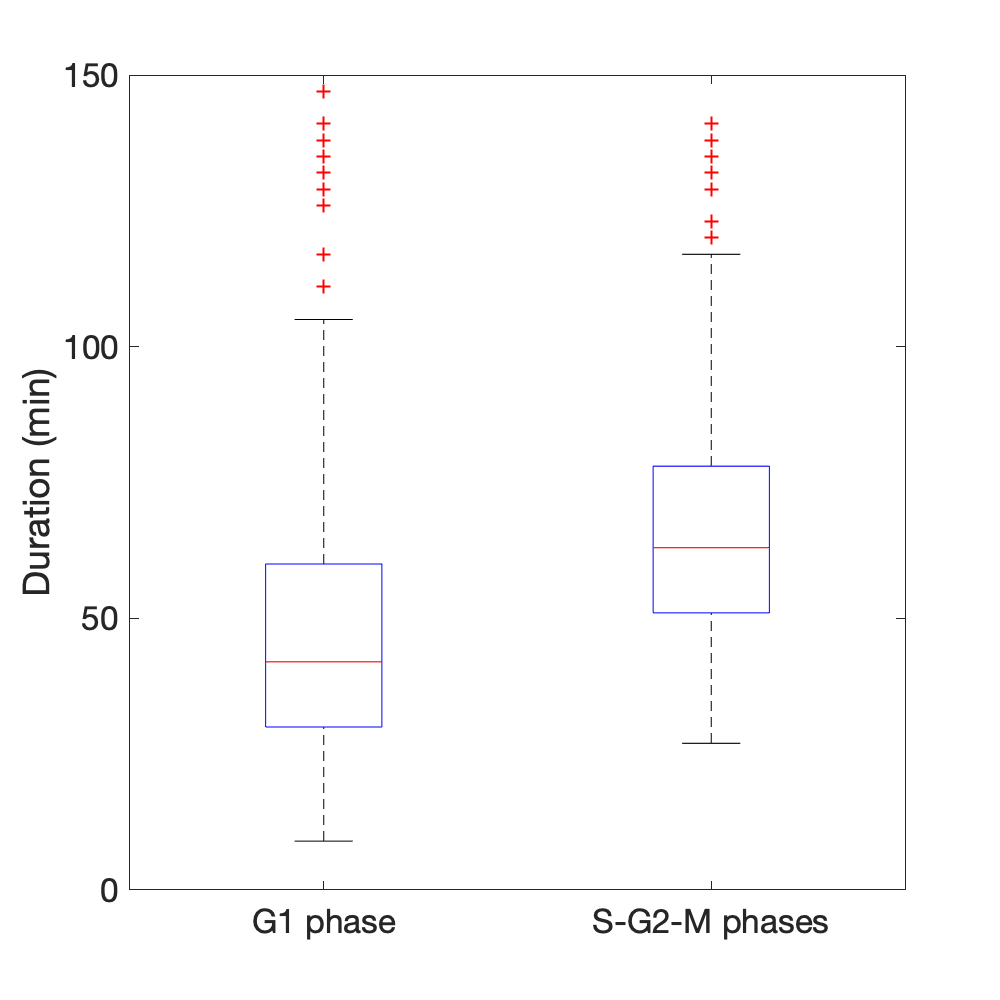

Supplement: Supplementary file 4 — Supplementary Data 1 [file 41467_2021_22689_MOESM4_ESM.zip › Supplementary_Data_1/Supplementary_Data_1.png]
