# Supplementary material for: Automatic synchronisation of the cell cycle in budding yeast through closed-loop feedback control
Source: Nat Commun. 2021 Apr 27;12:2452. doi: 10.1038/s41467-021-22689-w (PMC8079375; doi:10.1038/s41467-021-22689-w)
Supplement: Supplementary file 11 — Reporting Summary [file 41467_2021_22689_MOESM11_ESM.pdf]

nature research

Corresponding author(s): Diego di Bernardo

Last updated by author(s): Mar 22, 2021

Reporting Summary

Nature Research wishes to improve the reproducibility of the work that we publish. This form provides structure for consistency and transparency in reporting. For further information on Nature Research policies, see our Editorial Policies and the Editorial Policy Checklist.

Statistics

For all statistical analyses, confirm that the following items are present in the figure legend, table legend, main text, or Methods section.

n/a	Confirmed	
☐	☒	The exact sample size (*n*) for each experimental group/condition, given as a discrete number and unit of measurement
☐	☒	A statement on whether measurements were taken from distinct samples or whether the same sample was measured repeatedly
☒	☐	The statistical test(s) used AND whether they are one- or two-sided<br>*Only common tests should be described solely by name; describe more complex techniques in the Methods section.*
☒	☐	A description of all covariates tested
☒	☐	A description of any assumptions or corrections, such as tests of normality and adjustment for multiple comparisons
☐	☒	A full description of the statistical parameters including central tendency (e.g. means) or other basic estimates (e.g. regression coefficient) AND variation (e.g. standard deviation) or associated estimates of uncertainty (e.g. confidence intervals)
☒	☐	For null hypothesis testing, the test statistic (e.g. *F*, *t*, *r*) with confidence intervals, effect sizes, degrees of freedom and *P* value noted<br>*Give P values as exact values whenever suitable.*
☒	☐	For Bayesian analysis, information on the choice of priors and Markov chain Monte Carlo settings
☒	☐	For hierarchical and complex designs, identification of the appropriate level for tests and full reporting of outcomes
☐	☒	Estimates of effect sizes (e.g. Cohen's *d*, Pearson's *r*), indicating how they were calculated

Our web collection on statistics for biologists contains articles on many of the points above.

Software and code

Policy information about availability of computer code

Data collection	Raw microscopy images were acquired using Nis-Elements Advanced Research software v3.22.14 . Data were collected from raw microscopy images using custom MATLAB scripts available at https://github.com/dibbelab/Cycloop.
Data analysis	MATLAB R2019a was used to analyse data and make figures using a custom code provided at https://github.com/dibbelab/Cycloop.<br>All the simulations and the model were run using custom MATLAB scripts available at https://github.com/dibbelab/Cycloop/tree/main/Simulator.<br>The illustrative code of the stop&go controller is available at https://github.com/dibbelab/Cycloop/tree/main/Illustrative_Platform_Code/StopAndGo. Please note that the previous code also contains the phase estimation algorithm for the non-cycling strain.<br>The illustrative code of the reference oscillator controller is available at https://github.com/dibbelab/Cycloop/tree/main/Illustrative_Platform_Code/Reference_Oscillator. Please note that the previous code also contains the phase estimation algorithm for the cycling strain.

For manuscripts utilizing custom algorithms or software that are central to the research but not yet described in published literature, software must be made available to editors and reviewers. We strongly encourage code deposition in a community repository (e.g. GitHub). See the Nature Research guidelines for submitting code & software for further information.

Data

Policy information about availability of data

All manuscripts must include a data availability statement. This statement should provide the following information, where applicable:

- Accession codes, unique identifiers, or web links for publicly available datasets
- A list of figures that have associated raw data
- A description of any restrictions on data availability

The authors declare that the data supporting the findings of this study are available within the paper and its Supplementary Information files. Source data for Figs. 2-4 and Supplementary Figs. 1-9 are provided at https://github.com/dibbelab/Cycloop. The microscopy data have been deposited in Zenodo, https://doi.org/10.5281/zenodo.4516319.

Field-specific reporting

Please select the one below that is the best fit for your research. If you are not sure, read the appropriate sections before making your selection.

[x] Life sciences [ ] Behavioural & social sciences [ ] Ecological, evolutionary & environmental sciences

For a reference copy of the document with all sections, see nature.com/documents/nr-reporting-summary-flat.pdf

Life sciences study design

All studies must disclose on these points even when the disclosure is negative.

Sample size	Each experiment was repeated at least three times (n = 3) and is on par with standard experiments reported in the field.
Data exclusions	No data were excluded.
Replication	All attempts to replication were successful. Each control experiment was repeated independently at least three times with similar results.
Randomization	Randomization was performed thanks to the loading method that randomly filled the cells in the microfluidics device before each control experiment.
Blinding	Researchers were not blinded during experiments because the experiments were not based on qualitative scoring metrics.

Reporting for specific materials, systems and methods

We require information from authors about some types of materials, experimental systems and methods used in many studies. Here, indicate whether each material, system or method listed is relevant to your study. If you are not sure if a list item applies to your research, read the appropriate section before selecting a response.

Materials & experimental systems

n/a	Involved in the study	
[x]	[ ]	Antibodies
[x]	[ ]	Eukaryotic cell lines
[x]	[ ]	Palaeontology and archaeology
[x]	[ ]	Animals and other organisms
[x]	[ ]	Human research participants
[x]	[ ]	Clinical data
[x]	[ ]	Dual use research of concern

Methods

n/a	Involved in the study	
[x]	[ ]	ChIP-seq
[x]	[ ]	Flow cytometry
[x]	[ ]	MRI-based neuroimaging
